# Intravascular papillary endothelial hyperplasia of the spleen in a child: a case report

**DOI:** 10.1186/s12880-022-00936-w

**Published:** 2022-11-24

**Authors:** Wen-peng Huang, Li-ming Li, Li-na Zhu, Jian-bo Gao

**Affiliations:** Department of Radiology, The First Affiliated Hospital of Zhengzhou University, No.1, East Jianshe Road, Zhengzhou, 450052 Henan Province China

**Keywords:** Intravascular papillary endothelial hyperplasia, Spleen, Children, Ultrasound, Magnetic resonance imaging, Case report

## Abstract

**Background:**

Intravascular papillary endothelial hyperplasia (IPEH) is a vascular tumor characterized by the proliferation of endothelial cells with papillary formation. It is a rare benign condition affecting the head and neck. Currently, no cases of IPEH of the spleen have been reported. Here, we report a case of IPEH of the spleen in a child and discuss its clinical manifestations, imaging features, and surgical treatment.

**Case presentation:**

A 5-year-old female presented with a 4-month-old tumor in the left upper abdomen, abdominal pain, and constipation. She underwent radiography, barium enema, US, and MRI. A solid space-occupying mass was found in the left abdominal cavity on preoperative imaging, and it was diagnosed as angiosarcoma. The lesion was surgically resected. Histopathological analysis was consistent with IPEH.

**Conclusion:**

Clinicians should consider the possibility of IPEH in patients presenting with tumors in the spleen, which is curable by surgical resection. Malignant vascular tumors must be excluded in the differential diagnosis of IPEH to prevent misdiagnosis and inappropriate overtreatment.

## Background

Intravascular papillary endothelial hyperplasia (IPEH) is a rare, benign vascular lesion characterized by the reactive proliferation of endothelial cells. It is accompanied by papillary structures associated with thrombi and is considered to be the result of endothelial cell proliferation after thrombus recanalization. IPEH has been reported to account for approximately 2% of vascular tumors [1, 2]. It was first proposed by Pierre Masson in 1923, when he thought it was a type of vascular endothelial cell proliferation with a tumor-like nature; thus, it is also known as Masson's pseudoangiosarcoma [3]. IPEH can occur at any age and is more common among women [4]. It occurs in the skin of the head, neck, trunk, and cheek, as well as in the subcutaneous tissue of the nasal cavity, oral cavity, pharynx, larynx, and digestive tract [1–5]. Diagnosis is confirmed by histopathological analysis. To the best of our knowledge, IPEH of the spleen has not been reported in the English-language medical literature. IPEH of the spleen is rarer in children than in adults. Clinicians should consider the possibility of its occurrence in the spleen, its usually benign nature, and its treatment via surgical resection.

## Case presentation

**Fig. 1:**
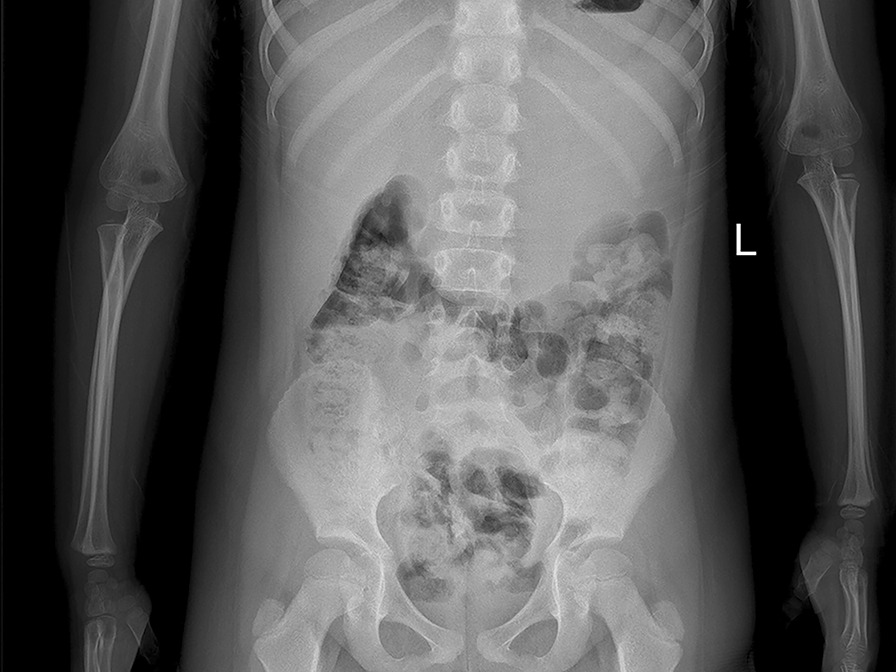
Digital radiography shows obvious accumulation of gases in the colon and no obvious abnormalities in the rest of the intestines

**Fig. 2:**
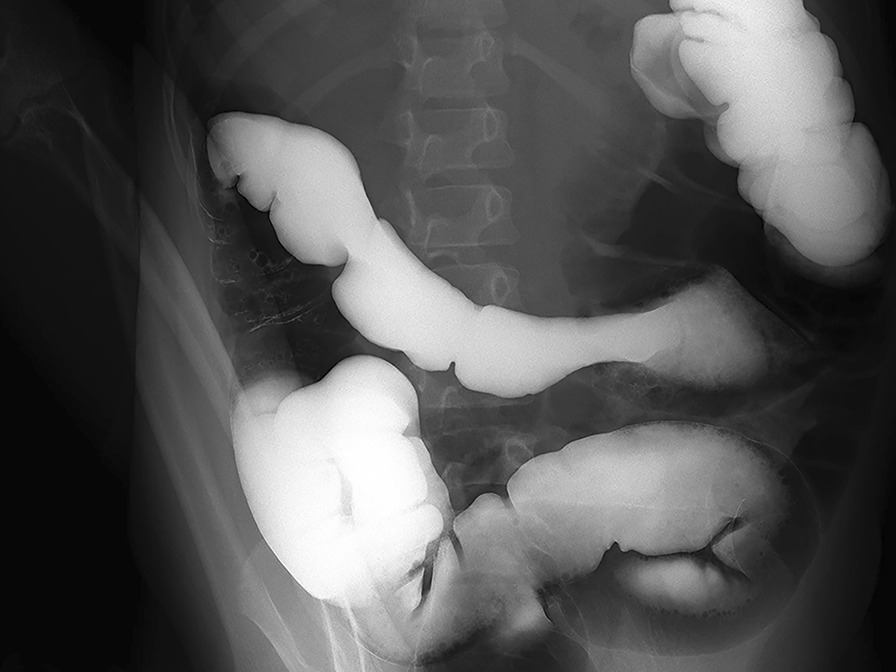
Barium enema shows a complete and clear colonic zone, no filling defect or abnormal stenosis, and a long transverse and sigmoid colon

**Fig. 3:**
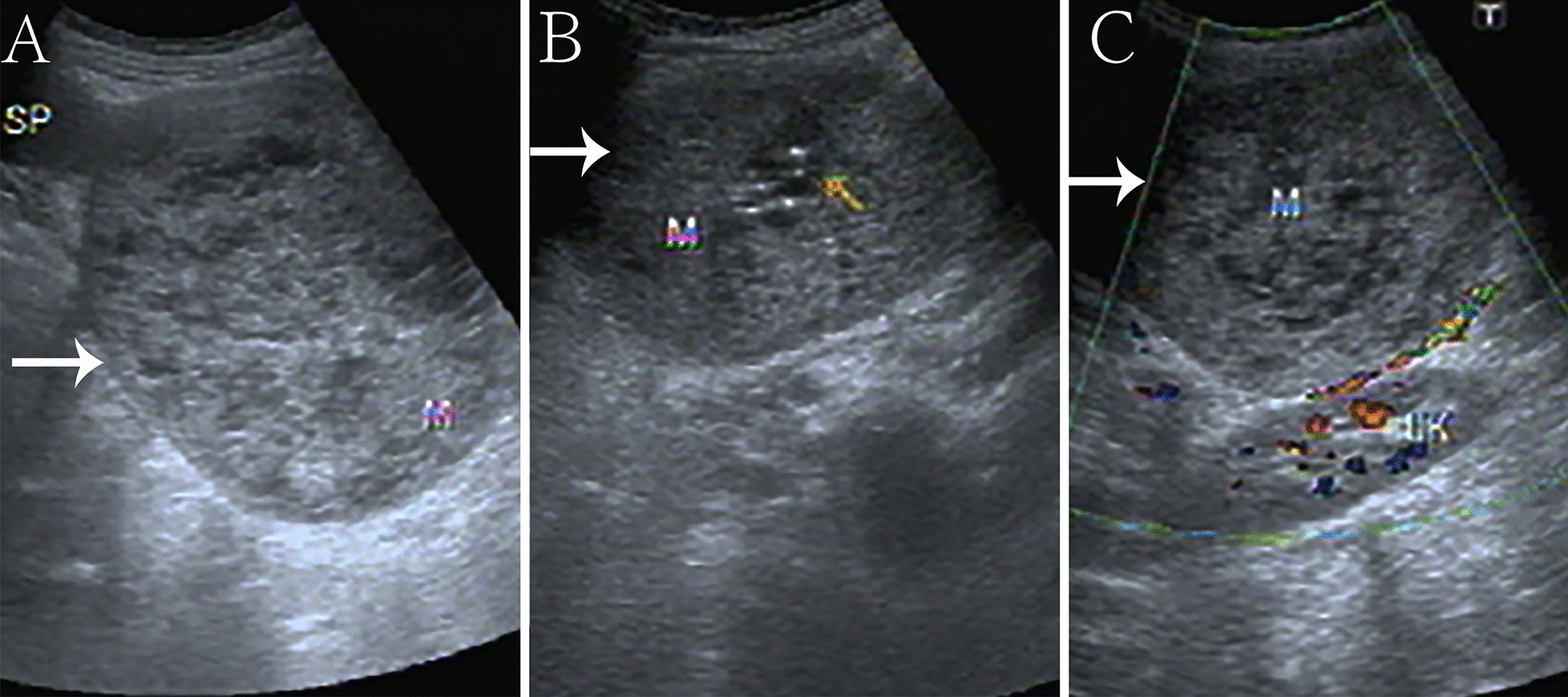
Grayscale and Doppler ultrasonography show a roundish heterogeneous mass below the spleen in the middle and lower abdomen on the left, with well defined margins, heterogeneous echotexture, with punctate echogenic foci, no acoustic shadowing (**A**, **B**). Doppler revealed multiple foci of vascularity within the mass, which also displayed peripheral vascularity (**C**)

**Fig. 4:**
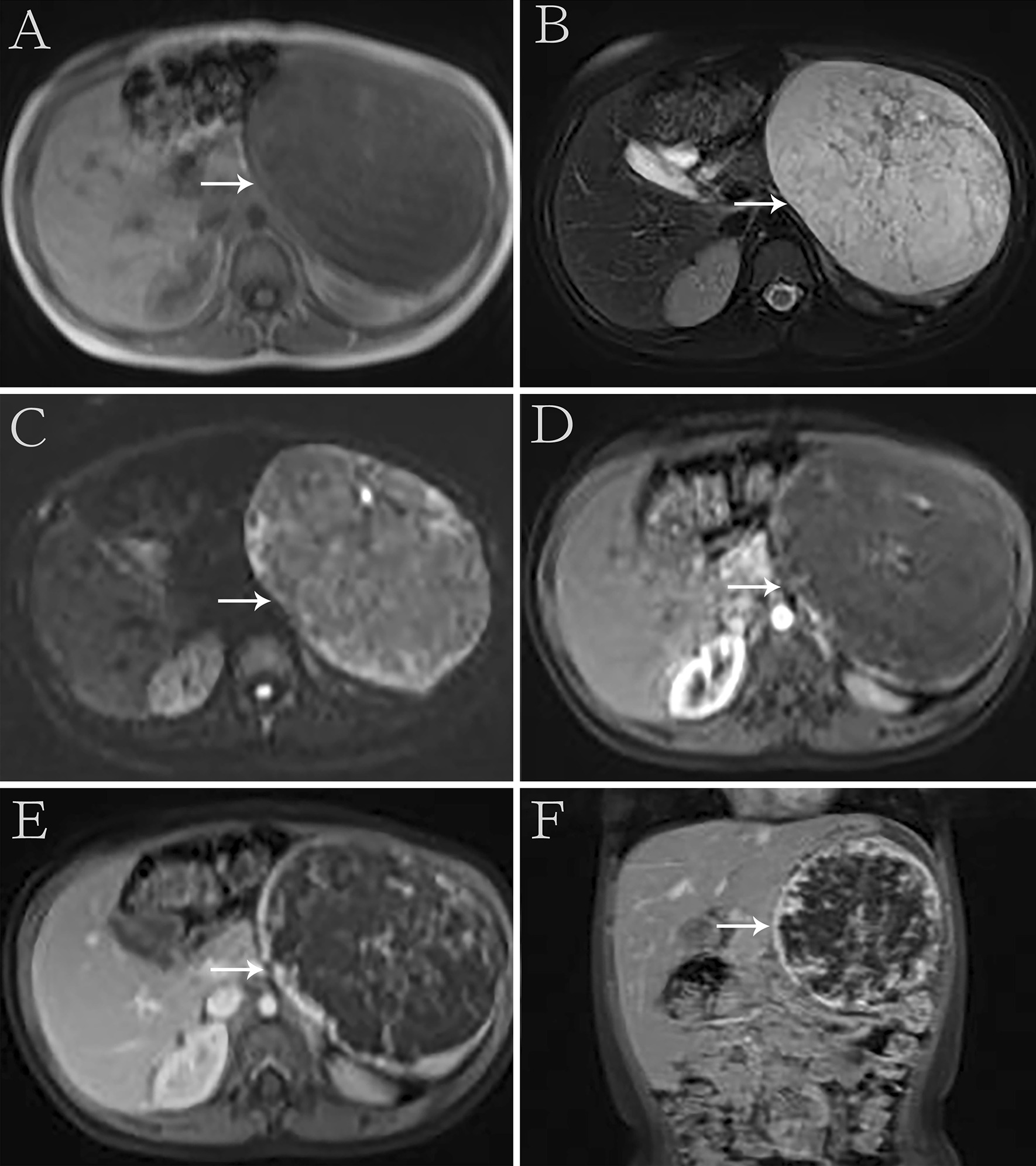
Magnetic resonance imaging shows the mass was globally hypointense on T1 weighted images, with some foci of increased signal intensity (**A**). Mildly heterogeneous, with predominantly high signal and some internal elongated (or linear) hypointense structures are shown on the T2-weighted images (**B**). Diffusion weighted images (b = 1000 s/mm^2^) demonstrated a heterogeneous pattern of increased diffusion, more prominent in the periphery (**C**). Multiple dilated and tortuous nutrient arteries were observed in the enhanced scanning arterial phase (**D**). Multiple foci of enhancement, with flaky appearances were observed in the portal phase (**E**). In the delayed period, continuous enhancement is seen in the lesions, with obvious enhancement at the edge (**F**)

A 5-year-old female visited our hospital with a palpable mass in her left upper abdomen for 4 months. She developed abdominal pain 4 months prior to the consultation. However, she did not undergo any treatment until the tumor increased in size, which prompted her visit to our hospital. Her past history, personal history, and family history were unremarkable. Physical examination revealed a mass in the left upper abdomen. The mass measured approximately 9.5 cm × 9.2 cm and had a hard texture. It was fixed and was not tender on palpation. The patient had no abnormal bowel sounds (heard four times per minute). Since the onset of the symptoms, the patient was conscious and oriented, and no remarkable changes were noted in diet, sleep, or urination. However, constipation was noted, with defecation occurring once every three days. She reported no weight loss. Laboratory test results showed normal levels of tumor-associated antigen (0.60 U/mL, normal range = 0.01–37 U/mL), carcinoembryonic antigen (4.69 ng/mL, normal range = 0–5 ng/mL), and alpha fetoprotein (3.58 ng/mL, normal range = 0–10 ng/mL). Abdominal radiography showed obvious accumulation of gases in the colon, but no obvious abnormality was found in the rest of the intestinal tract (Fig. 1). Subsequent barium enema revealed adequate filling and development of each segment of the colon under the double contrast of air and barium. Additionally, the colon was complete and clear; no filling defect or abnormal strictures were found. The transverse and sigmoid colon were long, and no obvious stricture was found in the anal canal (Fig. 2). Further ultrasonography (US) revealed a round heterogeneous mass of size 11.6 cm × 9.7 cm in the left upper and mid quadrants with well defined margins, heterogeneous echotexture, with punctate echogenic foci, no acoustic shadowing. The mass was close to the superior mesenteric artery, which showed no abnormalities. Doppler revealed multiple foci of vascularity within the mass, which also displayed peripheral vascularity (Fig. 3). To further determine the nature of the lesion, the patient underwent abdominal magnetic resonance imaging (MRI). A 11.2 cm × 11.1 cm × 10.8 cm (LR × AP × SI) mass was identified in the left upper quadrant. The mass was globally hypointense on T1 weighted images (T1WI), with some foci of increased signal intensity. On T2 weighted images (T2WI), the mass was mildly heterogeneous, with predominantly high signal and some internal elongated (or linear) hypointense structures. Diffusion weighted images (b = 1000 s/mm^2^) demonstrated a heterogeneous pattern of increased diffusion, more prominent in the periphery, with diffusion restriction on ADC maps. The margin of the lesion was well defined, and a surrounding capsule was noted. There was obvious compression and displacement of the stomach, spleen, and left kidney. The left adrenal gland was not clearly displayed. The head of the pancreas was clearly displayed; however, the tail of the pancreas was not. Multiple dilated and tortuous nutrient arteries were observed in the enhanced scanning arterial phase, multiple foci of enhancement, with flaky appearances were observed in the portal phase, and continuous enhancement was observed in the delayed phase, with obvious enhancement at the edge (Fig. 4). The radiologist considered the abdominal mass to be splenic hemangioma or angiosarcoma.

**Fig. 5:**
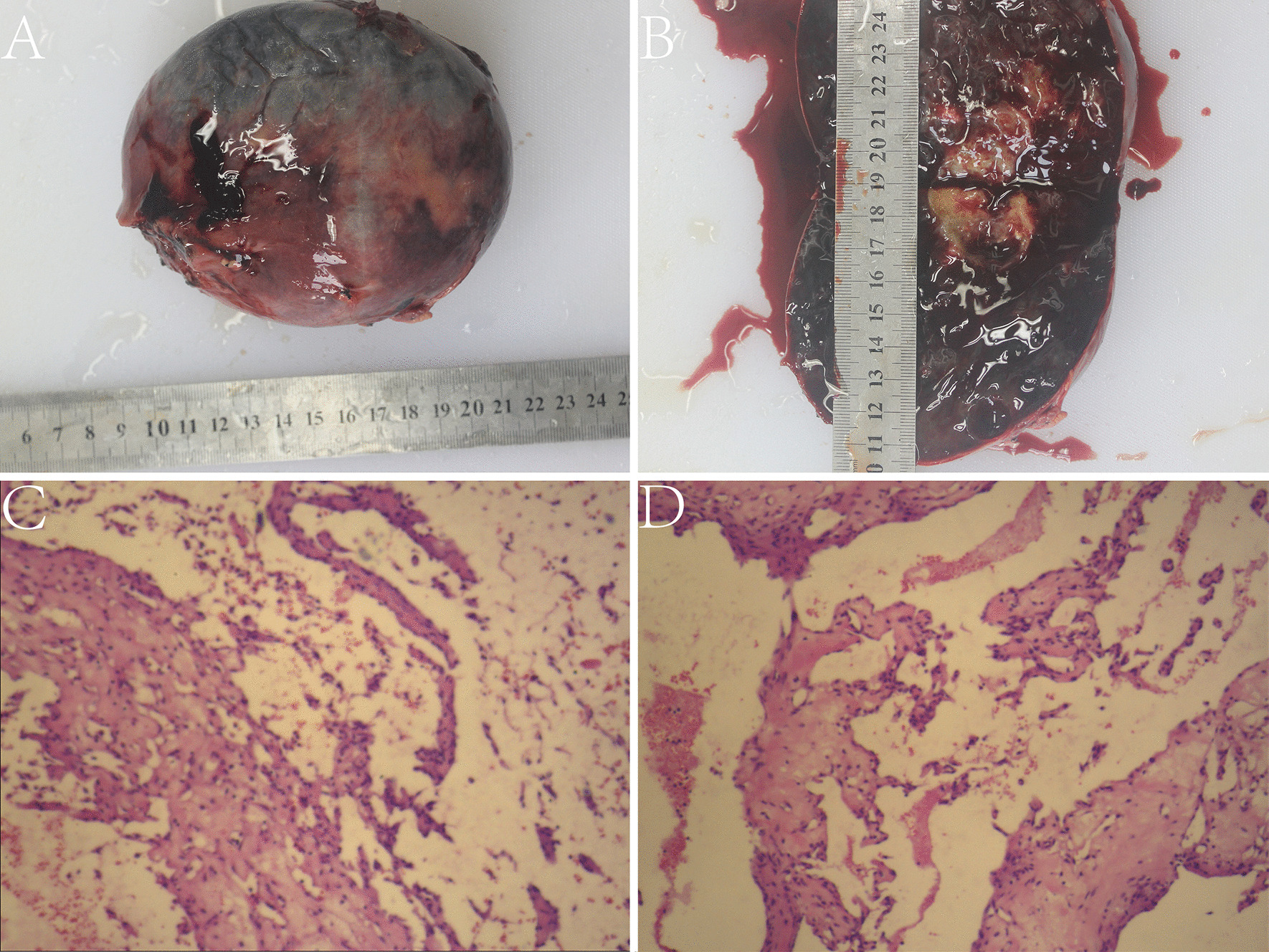
Gross specimen image and optical microscopy image of intravascular papillary endothelial hyperplasia shows gray–red tissue of 12.0 cm × 10.0 cm × 6.5 cm in size. The surface is smooth, and the capsule is intact (**A**). The section is grayish red and brown, and some areas are gray and white (**B**). The vascular endothelial cells are fusiform and form multiple papillary processes (**C**). The lesion is full of anastomosed fissure vascular lumen and scattered organized thrombus (**D**)

After general anesthesia, the patient underwent splenectomy. Under the left costal margin, the skin, subcutaneous fascia, and each layer of the rectus abdominis muscle were dissected and opened, and the peritoneum was cut to enter the abdominal cavity. The tumor was located on the outside of the descending colon. The boundary between the upper end of the tumor and the spleen was unclear, and the lower end was located in the upper pole of the left kidney. The surface was covered by the greater omentum, and several nutrient vessels were seen penetrating the tumor. The tumor was spherical, smooth, and approximately 12 cm in diameter. Splenic hemangioma was suspected before the operation, and most splenic hemangiomas in children are capillary hemangiomas. However, during the operation, it was found that more nutrient vessels penetrated into the tumor. This finding is not consistent with the manifestation of splenic hemangiomas in surgery, but it is consistent with angiosarcomas. The surface tissue of the omentum was carefully dissected, the nutrient vessels were excised, and the tumor was completely removed. Hemostasis was achieved, and the wound was closed using biological hemostatic paper and fibrin glue. There was no active bleeding in the wound. No injury was noted to the hilum of the spleen, and good blood supply to the spleen was noted. Histopathology after surgery showed erythrocytes, fibrous tissue, hyperplasia of small vessels, spindle-shaped vascular endothelial cells, lesions filled with anastomosing lacunae and scattered mechanized thrombi (Fig. 5). Immunohistochemical staining was positive for CD31, CD34, SMA, ERG, and negative for EMA, PR, GFAP, Desmin, corresponding to the phenotypic profile of IPEH. After the operation, the surgical incision had healed well without redness, swelling, or inflammatory secretions, and the patient did not complain of any discomfort. The patient’s family members requested her discharge from the hospital after 2 weeks. She was reviewed every 6 months. Follow-up over 3 years showed no recurrence or other complications.

## Discussion

IPEH is a vascular lesion that shows reactive proliferation of endothelial cells in the organizing thrombus. Based on the participation of endothelial cell proliferation around the thrombus during venous stasis, IPEH can be classified into three different types. The first type, which accounts for approximately 56% of all cases, is present in the dilated vascular space or in the lumen of pre-existing vascular lesions, usually veins. The second type is the mixed type, which accounts for approximately 40% of all cases, and the lesions are secondary to the original vascular lesions, such as arteriovenous malformations, hemangiomas, varicose veins, and suppurative granulomas. The third type, which is relatively rare, accounts for approximately 4% of all cases [6, 7] and is an extravascular type associated with hematoma tissue. In this study, the patient was born healthy and had no primary vascular disease or related family history. Her condition was classified as the primary type. The clinical manifestations of IPEH are not specific in most patients [4]. In this case, the lesion was located in the spleen and manifested as a mass in the left upper abdomen. The patient presented with abdominal pain and constipation. No obvious abnormality was found on abdominal radiograph or barium enema. Abdominal pain and constipation might have been caused by excessive compression of the intestines and adjacent organs.

Although the etiology and pathogenesis of IPEH is unclear, several mechanisms have been proposed. Some scholars believe that the process of thrombosis attracts macrophages and promotes endothelial cell proliferation through the release of basic fibroblast growth factor. This process initiates a vicious cycle of the secretion of basic fibroblast growth factors and promotion of endothelial cell proliferation [4]. Based on the characteristics of thrombosis, some researchers believe that IPEH may be closely associated with blood stasis at the thrombosis site and intimal inflammation caused by local hemodynamic changes [8]. Some researchers believe that IPEH involves primary vascular endothelial cell hyperplasia followed by hemodynamic changes, which further lead to thrombosis [9]. Histopathological examination is crucial for the definite diagnosis of IPEH [5]. The typical histological features of IPEH include multiple papillary processes that are formed by noninvasive proliferating endothelial cells, which protrude into the vascular lumen [2]. The core of the papillary structure is transparent cellulose that forms a fissure-like vascular lumen with irregular anastomosis and communication and is connected to the thrombus. Monocyte infiltration can be seen in the surrounding matrix. The endothelial nucleus is uniform and slightly pleomorphic at high magnification. A residual thrombus is often observed in the lesions. IPEH is usually positive for CD31 and CD34, which are the most sensitive markers indicating the origin of diseased blood vessels [4]. The main pathological problem may be its similarity with malignant vascular tumors, such as angiosarcoma or Kaposi’s sarcoma. However, the intravascular location and the presence of papillary structures supported by the core of the thrombus, normal mitotic figures, and lack of cellular atypia are the histological characteristics that can be considered for the differential diagnosis [10].

IPEH lesions appear circumscribed with variable density on computed tomography (CT), hypo-to iso-intensity on T1WI, and iso- to hyperintensity on T2WI with obvious enhancement [1]. In this case, the US features of the spleen IPEH showed a well-defined, round heterogeneous mass with uneven internal echoes, strong, dotted echoes and no sound behind. The mass was close to the superior mesenteric artery, which was not abnormal, suggesting that the lesion originated from the venous system. Doppler revealed an internal punctate blood flow signal surrounded by a peripheral blood flow signal, which might have been due to the large number of hyperplastic blood vessels in the area around the lesion. The punctate echogenic foci in the lesion is considered as sclerotic thrombus tissue. MRI showed mixed low signal intensity of T1WI and mixed high signal intensity of T2WI. The signal was mixed because old hemorrhages and infarcts are often present in the lumen of IPEH, and a residual sclerotic thrombus is often seen in these lesions. The mixed T2WI signal may reflect the different phases of thrombosis. The enhancement mode and characteristics of IPEH are related to the histopathology and distribution of supply vessels. These findings are consistent with the formation of multiple papillary structures formed by vascular endothelial cell proliferation and the formation of a vascular network, which may have significance for the differential diagnosis of soft tissue tumors.

In this case, patchy low intensity was seen in the lesion, which was considered organized thrombus tissue. Additionally, the edge of the lesion was obviously enhanced, which might have been caused by the fibrous pseudocapsule formed by hemosiderin deposition around the IPEH.

IPEH should be distinguished from hemangioma, hamartoma, primary lymphoma, and angiosarcoma of the spleen. On MRI, hemangiomas are either hypo- or isointense on T1WI and heterogeneously hyperintense on T2WI. A high-signal intensity on T1WI suggests the presence of subacute hemorrhage or proteinaceous content. After enhanced scanning, hemangiomas usually demonstrate early peripheral nodular enhancement with continuous filling-in that is homogeneous on delayed scanning [11]. Plain CT of the splenic hamartoma showed well-defined low density, few internal calcifications, and a low signal or mixed low signal on T1WI on MRI. When a high signal was observed in the lesion, fat-saturated T2WI was used to determine the presence of fat components. T2WI was heterogeneous with slightly high signal intensity, and an enhanced scan of early lesions showed diffuse heterogeneous patchy enhancement and fat components with no enhancement [12]. Primary splenic lymphoma has a hypointense signal on T1WI and a varied signal appearance on T2WI. Moreover, diffuse lymphoma shows splenomegaly with irregular high or low signal areas, while mass lymphoma shows low signal lesions in the spleen with a high-signal intensity [13]. Splenic angiosarcoma is a rare invasive tumor. Plain CT shows increased heterogeneity of the spleen, low density, and unclear boundaries. After contrast-enhanced scanning, the tumor was enhanced from the edge of the lesion and gradually filled the center. T1WI shows low signal intensity, and T2WI shows an obvious high-signal intensity on MRI. The enhanced scan is the same the CT scan [14]. It is important to exclude angiosarcoma to prevent clinical misdiagnosis and inappropriate overtreatment. IPEH is a benign disease that is mainly treated by the complete removal of the mass via surgery, and it has a good postoperative prognosis. At present, no cases of metastasis or recurrence have been reported [4, 5]. Some scholars have proposed that radiotherapy can reduce the volume of residual tumors and may provide more lasting local control [15]. In this case, through complete surgical resection, the prognosis of the patient was good. Considering the adverse effects of radiotherapy on child development, it was not used.

## Conclusion

In summary, the clinical manifestations of IPEH are very varied, as they differ in size, shape, internal structure, and growth pattern of the lesion. Definitive diagnosis depends on pathological examination, and imaging can be used as an auxiliary tool for diagnosis. It is necessary to continuously report new IPEH cases, as it can improve our understanding of its clinical and imaging features. Clinicians should be aware of the possibility of its occurrence in the spleen, its usually benign nature, and its treatment by surgical resection.

## Data Availability

All data generated or analyzed during this study are included in this published article.

## Abbreviations

IPEH: Intravascular papillary endothelial hyperplasia
MRI: Magnetic resonance imaging
T1WI: T1-weighted images
T2WI: T2-weighted images
US: Ultrasonography
CT: Computed tomography
